# An observational study of adult admissions to a medical ICU due to adverse drug events

**DOI:** 10.1186/s13613-016-0109-9

**Published:** 2016-02-02

**Authors:** Pierre-Alain Jolivot, Claire Pichereau, Patrick Hindlet, Gilles Hejblum, Naïke Bigé, Eric Maury, Bertrand Guidet, Christine Fernandez

**Affiliations:** Sorbonne Universités, UPMC Univ Paris 06, INSERM, Institut Pierre Louis d’Epidémiologie et de Santé Publique (IPLESP UMRS 1136), Equipe 6, 75012 Paris, France; APHP, Hôpital Saint-Antoine, Service de Pharmacie, 75012 Paris, France; APHP, Hôpital Saint-Antoine, Service de Réanimation Médicale, 75012 Paris, France; Univ Paris-Sud, Faculté de Pharmacie, 92296 Châtenay-Malabry, France

**Keywords:** Drug-related side effects and adverse reactions, Medication errors, Medication adherence, Self-medication, Intensive care units, Incidence

## Abstract

**Background:**

The objectives of the study were to estimate the incidence of intensive care unit (ICU) admissions due to adverse drug events (ADEs), to assess preventability, severity and costs of the corresponding ADE and to determine the leading causes of preventable ADEs.

**Methods:**

An observational study was conducted in a medical ICU of a teaching hospital from February 2013 to February 2014.

**Results:**

A total of 743 consecutive admissions were included, and they involved 701 different patients. The included admissions were categorized into three groups (admissions due to preventable ADE, admissions due to unpreventable ADE and the control group). Among the 743 ICU admissions included during the study period, 173 (23.3 %) were due to ADE, with 102 (13.7 %) related to preventable ADE and 71 (9.6 %) to unpreventable ADE, yielding a preventability rate of ADE of 0.59 (102/173). Admissions due to unpreventable ADE concerned patients with more comorbidities, a greater number of drugs and higher Simplified Acute Physiology Score II than admissions due to preventable ADE and the control group admissions (*n* = 570). Hospital mortality rates, corresponding costs and length of stay were all similar in the preventable ADE and control groups, whereas they were always significantly higher in the unpreventable ADE group. ICU mortality, length of stay and the corresponding costs were similar in the three groups. Non-compliance was the principal leading cause of preventable ADE (*n* = 31/102). The 102 preventable ADE-related admissions accounted for a total of 528 days of hospitalization in the ICU, requiring a mean of 1.4 ICU beds per day over the one-year period, with an associated total cost amounting to 747,651 €.

**Conclusions:**

ADE was a major cause of admission in the studied ICU, and in 59 % of the cases, ADEs were preventable. The reported burden of ICU admissions due to ADE advocates for further investigations to explore how the rate of such admissions could be decreased.

**Electronic supplementary material:**

The online version of this article (doi:10.1186/s13613-016-0109-9) contains supplementary material, which is available to authorized users.

## Background

Drug-related problems are a significant burden for healthcare facilities as they account for 5.3–12.1 % of hospital admissions, depending on the studies and the definition used for an adverse drug event [1, 2]. For instance, adverse drug events (ADEs) were defined by Nebeker et al. [3] as “any injury from medical intervention related to a drug.” This broad definition encompasses unpreventable ADEs also called adverse drug reactions (ADRs) and preventable ADEs, resulting from medication errors (ME).

An ADR was defined by the World Health Organization (WHO) as “any noxious and unintended effect of a drug occurring at doses normally used in man for the prophylaxis, diagnosis, or therapy of the disease, or for the modification of physical function” [4]. Finally, ME, as opposed to ADR, can be defined as “any preventable events that may cause or lead to inappropriate medication or patient harm while the medication is in the control of the healthcare professional, patient or consumer” [3]. Preventable events include drug overuse, underuse and misuse as defined by the American National Roundtable on Health Care Quality [5].

Patients hospitalized for an ADE have longer hospital length of stay (LOS) and higher risks of death than other patients [6]. Admissions into the intensive care unit (ICU) deserve special attention since they account for most severe admission cases with a potential fatal threat. However, data related to ADE as a cause of ICU admission are scarce and heterogeneous. Based on 12 studies published between 1986 and 2014 [7–18], a recent systematic review reported incidences of ICU admissions due to an ADE ranging from 0.37 to 27.4 % and mortality rates ranging from 2 to 28.1 % [7–19]. Preventable ADEs accounted for 17.5 to 85.7 % of the ICU admissions. Many features contributed to the studies’ heterogeneity, including case mix, ADE definitions, methods used for assessing causality and preventability. Moreover, the leading causes of preventable ADE were poorly investigated [7, 8, 10, 12, 13]. It is important to note that the implication of non-compliance or self-medication in ICU admissions was not explored in these studies. Finally, the costs of ICU admissions due to ADE and of LOS were seldom assessed [10, 17]. Therefore, we undertook a study examining in detail the critical issues related to the admissions to ICU related to an ADE. Based on the admissions observed in a medical ICU during a one-year period, our main objective was to determine the incidence of ICU admissions due to an ADE. We also identified which admissions were related to a preventable ADE and determined the leading causes of the corresponding ADE, and which were related to an unpreventable ADE. Finally, the severity of patients’ condition, the LOS and the associated costs were compared in the three groups of admissions considered: preventable ADE-attributed admissions, unpreventable ADE-attributed admissions and the control group.

## Methods

### Study design and setting

This observational monocentric study was conducted over a one-year period, from February 24, 2013, to February 23, 2014, in a 18-bed medical ICU of a university-affiliated 760-bed hospital (Saint-Antoine Hospital, AP-HP, Paris, France). Saint-Antoine hospital is a multidisciplinary hospital with specialization in hepato-gastroenterological and onco-hematological diseases and a solid tumor and hematopoietic stem cell transplantation expertise. The hospital is also equipped with a digestive surgical ICU (the present study was not conducted in the latter unit). Patients were followed up from their admission in the ICU through to their discharge from the hospital.

### Participants

The inclusion criterion was the following: all ICU admissions occurring during the study period. Exclusion criteria were age under 18, patient refusal or impossibility to investigate the treatment or medical history and patients admitted and discharged during the same weekend. Patients admitted for external care (bronchoscopy, renal replacement therapy for chronic renal failure or central venous catheter insertion) were also excluded from the study. Readmissions were considered as new ICU admissions and analyzed accordingly.

The need for a written consent was waived since it was a study on usual care, without any specific intervention. All patients and relatives were informed that anonymous data could be used for academic research. The study was approved by an institutional review board (Commission d’Ethique de la Société de Réanimation de Langue Française, Paris, France).

### Admissions’ screening

Two investigators, a pharmacist (PAJ) and an ICU physician (CP), screened independently all ICU admissions during the morning staff meeting from Monday to Friday. After reviewing each medical chart, they independently sorted all included admissions into two groups: the ADE group (ICU admissions due to ADE) and the control group (ICU admissions for a matter other than ADE). In case of disagreement, BG (Professor of Intensive Care) or CF (Professor of Clinical Pharmacy) classified the admission as related or not to an ADE.

For both assessors, the causal relationship between drugs and clinical features was assessed according to chronological, semiological and bibliographical data. Chronological data included the chronology of the events (drug administration or interruption and clinical signs), assessment of drug exposure at the beginning of the first clinical signs, taking into account drug pharmacokinetics. Additionally, clinical consequences (recovering or not) after drug dechallenge/rechallenge were assessed when possible. Semiological data were based on determining the possible etiologies of the observed clinical signs and on specific laboratory test results. Bibliographical data search was mainly based on the Summary Product Characteristics (SmPC), and in the case of lack of information in the SmPC, additional sources of data were used (Micromedex^®^ and/or search in Embase database or MEDLINE database via PubMed).

ADEs were classified as preventable or unpreventable according to the Schumock and Thornton modified criteria (see the subsection Data sources/measurement—preventability in the “Methods” section) [20]. Preventable ADEs were subcategorized into 3 classes according to the cause of the event: drug overuse, underuse and misuse. Drug overuse stood for situations in which potential for harm exceeded the possible benefit. Drug underuse corresponded to failures to detect diseases or to use proven effective treatments. Drug misuse corresponded to an appropriate treatment with occurrence of a preventable complication [5]. Non-compliance and self-medication were considered as misuses.

ICU admissions related to non-compliance and self-medication were included in the ADE group, whereas admissions related to self-poisoning were included in the control group.

### Data sources/measurement

#### For all included ICU admissions

The following data, available in medical records, were collected for every included ICU admission: age, gender, comorbidities, patients’ origin (home or hospital), main reason for admission (according to the 10th International Classification of Diseases) [21] and LOS or vital status at ICU and hospital discharge. The Simplified Acute Physiology Score II [22] and the Sequential Organ Failure Assessment [23] were calculated within a period of 24 h after ICU admission. The McCabe score was used as an integrative index of the severity of underlying medical condition [24]. The need for and the number of organ supports were recorded (invasive or noninvasive mechanical ventilation, vasopressors, massive transfusion or renal replacement therapy).

Patient origin was considered as home if he/she had been admitted into the ICU through the French Emergency Medical Aid Unit or after visiting Emergency Department. In any other cases (i.e., transfer to the ICU from another unit or from another hospital), patient origin was considered as hospital.

In order to identify all drugs prescribed before admission and self-medication and to detect non-compliance, the patients and/or their relatives were questioned and medical records were analyzed. Whenever drug prescriptions were unavailable, general practitioners, specialists or pharmacists in charge of the patients were contacted. Whenever patients were hospitalized before ICU admission, the list of all drugs administered during the hospital stay was retrieved. A dichotomous categorization of drugs was considered according to the delay between ICU admission and drug prescription: either at most a month or more.

ICU and hospital costs are expressed in euros for the year 2013. These were the direct costs of the hospitalization adopting the perspective of the payer, i.e., the total fees of the admissions invoiced to the payer. Costs were directly issued from the administrative database of the hospital and are based on the French Diagnosis-Related Group system specially adapted to ICU admissions including the need for organ support [25].

#### Specifically for the ADE group

##### Drug classes and clinical features

For each drug suspected of being involved in an ADE, the investigators recorded the following data: route of administration, daily schedule, starting time and end of treatment, and biological parameters. Drug classes were coded according to the Anatomical Therapeutic and Chemical (ATC) classification system [26].

Main ADE-related organ failures were classified according to the System Organ Class (SOC) codes of Medical Dictionary for Regulatory Activities (MedDRA) version no. 18.0 (Additional file 1: Table S1).

##### Causality

Except for admissions due to non-compliance and lack of treatment, the relationship between a drug and an ADE was evaluated using 3 standardized causality assessment methods: the official French method [27] and 2 international methods, the Naranjo [28] and the Karch and Lasagna methods [29]. ICU admissions were allocated to the ADE group if a drug was very likely (or certain), likely (or probable) and possibly involved according to at least 2 out of 3 causality assessment methods. Whenever several drugs were involved in a single ICU admission, the strongest causality link was retained to characterize the admission.

##### Preventability

The preventability of ADE was assessed according to Schumock and Thornton [20] with an additive criterion (ADEs due to the lack of treatment were considered preventable) and a modified criterion (ADEs were considered preventable if the type of drug–drug interaction was contraindicated). Whenever several ADEs were involved in a single ICU admission, the admission was considered as unpreventable if at least one ADE was unpreventable.

Both above-mentioned investigators independently identified the leading causes of preventable ADE according to a pre-established list. In the case of several leading causes for a single admission, the principal leading cause was retained. The cause of admission was then subclassified as related to drug underuse, overuse or misuse.

Definitions used for ADE, ADR, ME, assessment methods for preventability and causality are presented in Additional file 2: Table S2.

### Bias

As described above, the sorting of patients into one of the two groups (ADE or control) was made according to 3 causality assessment methods. These methods are based on the literature data and on chronological, semiological and pharmacological analysis. Moreover, this sorting was assessed by two independent investigators who both intended the daily ICU medical staff.

### Study size

Study size was planned to obtain a reasonable confidence interval of the incidence of ICU admissions caused by ADE. Assuming such an incidence to be at 15 % (a rough estimate issued from a pilot experiment conducted in our ICU) and about 700 ICU admissions that would be included in the study in 1 year, the period of a one-year study was retained. According to Agresti and Coull method [30], such a study would a mean incidence estimate of [95 % confidence interval] 15 % [12.5; 17.9].

### Quantitative variables

Each drug of a chemotherapeutic combination was considered individually for the determination of the number of drugs taken before ICU admission. However, for the determination of the number of drugs involved in the ADE, the generic term “antineoplastic agents” was used and the chemotherapeutic combination was counted as one drug in the case of drug-induced neutropenia or tumor lysis syndrome. Indeed, these ADEs were considered as a consequence of a combination rather than of a specific antineoplastic agent.

The number of comorbidities, organ supports and drugs involved in the ADEs were categorized into four groups (0, 1, 2 and ≥3) and were analyzed as categorical variables.

Hospital LOS, mortality and costs were censured at discharge from Saint-Antoine hospital.

### Statistical methods

#### Statistical analyses

The incidence of ADE-related admissions was calculated as the ratio of the number ADE-related admissions to the total number of included ICU admissions.

Results on quantitative variables are presented as medians and inter-quartile ranges, and those on qualitative variables are presented as numbers and associated percentages. Group comparisons on quantitative and qualitative variables were performed with the Mann–Whitney and Wilcoxon test and the Fisher exact test, respectively. Since multiple comparisons involving 3 groups were performed, the threshold *P* value of 0.0166 was considered for statistical significance, according to the applied Bonferroni correction. Inter-rater agreement (ADE vs control group, 3 causality assessment methods and preventability assessment) was assessed with the kappa test [31]. Statistical analysis was performed with R Studio software (version 0.98.490).

#### Sensitivity analysis

As our study was focused on the burden of admissions caused by unintentional medication related problems, all other admissions including self-poisoning were put in the control group. Nevertheless, a sensitivity analysis excluding self-poisoning cases was conducted in order to (1) be consistent with previous studies which had excluded such cases [7, 11, 13, 14, 17] and (2) explore the impact of self-poisoning cases on the study results.

## Results

**Fig. 1:**
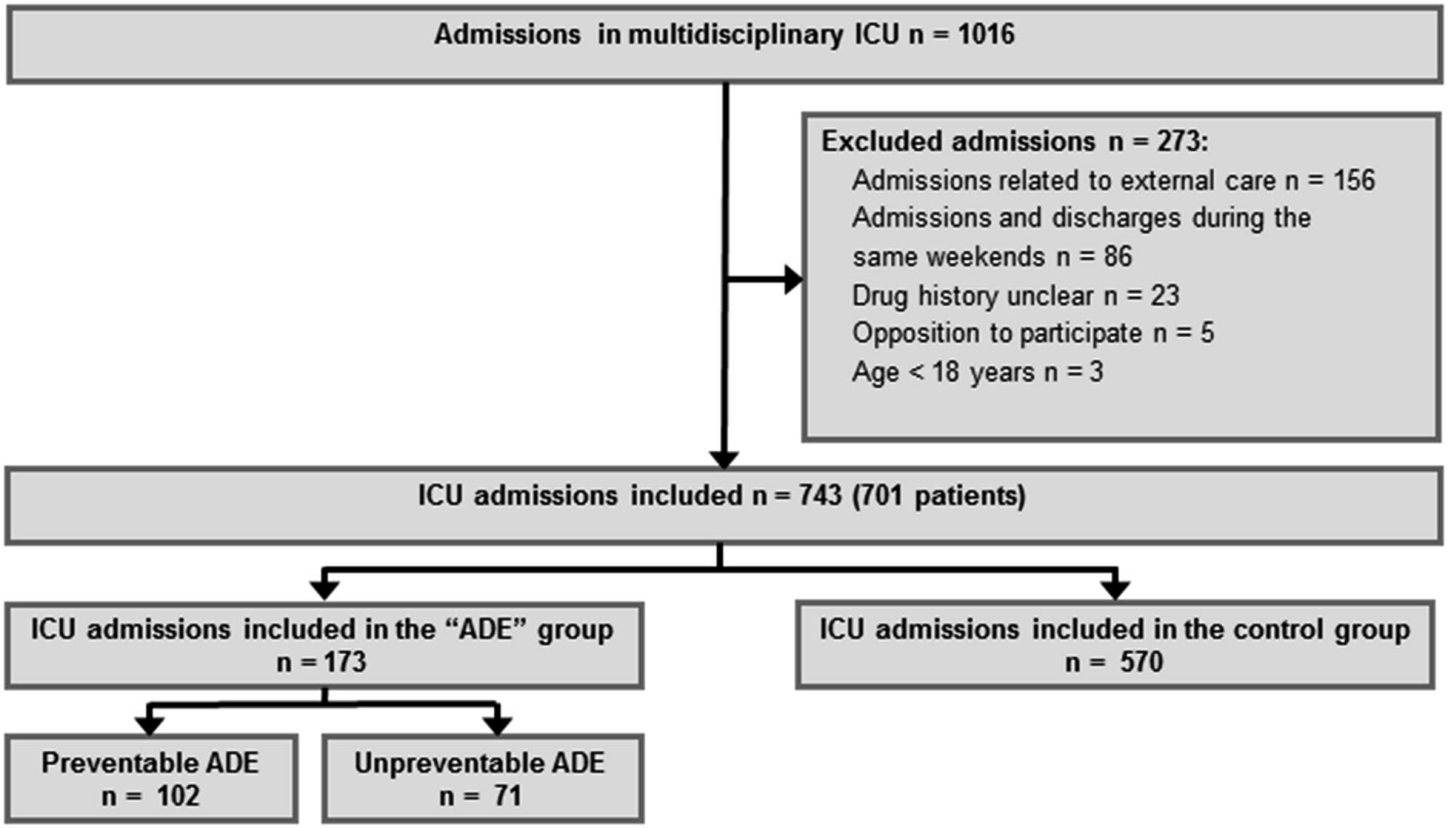
Flowchart of the study. *ICU* intensive care unit, *ADE* adverse drug event

There were 743 admissions included in the study out of the 1016 recorded ICU admissions (Fig. 1). Included admissions concerned 701 patients (33 patients were admitted twice, three patients were admitted three times, and one patient was admitted four times). There were 173 and 570 admissions finally categorized as ADE-related and control admissions (inter-rater agreement corresponding kappa test = 0.97), respectively, resulting in an incidence estimate of ADE-related admissions at 23.3 % [95 % confidence interval (CI) 20.4–26.5]. There were 71 ICU admissions attributed to unpreventable ADE (41.0 % [95 % CI 34.0–48.5] of ADE-related admissions or 9.6 % [95 % CI 7.6–11.9] of all ICU admissions) and 102 to preventable ADE (59.0 % [95 % CI 51.5–66.0] of ADE-related admissions or 13.7 % [95 % CI 11.4–16.4] of all ICU admissions). The corresponding inter-rater agreement was excellent (kappa test = 0.98).

Table 1 details the baseline characteristics of the patients at admission in the ICU in the three groups considered (preventable ADE-related group, unpreventable ADE-related group and control group). Age and gender did not significantly differ from one group to another. The main organ failures associated with each ATC class are summarized in Additional file 1: Table S1. The 173 ADE-related admissions were associated with 219 drugs which induced 219 organ failures (100 were in the unpreventable group and 119 in the preventable group).

**Table 1:** Baseline characteristics of the patients at admission in the intensive care unit

Characteristics	Total (*n* = 743)	Preventable ADE (*n* = 102)	Unpreventable ADE (*n* = 71)	Control (*n* = 570)	*P* value		
					Preventable–unpreventable	Preventable–control	Unpreventable–control
Age (median [IQR])	65 [51; 78]	63 [47; 77]	65 [55; 75]	65 [50; 78]	0.60	0.63	0.92
Gender (males/females; sex ratio)	427/316; 1.35	50/52; 0.96	43/28; 1.53	334/236; 1.42	0.16	8.2 × 10^−2^	0.80
Number of underlying disease(s) [*n* (%)]					1.2 × 10^−2^	5.7 × 10^−3^	6.7 × 10^−9^
0	95 (13 %)	5 (5 %)	0 (0 %)	90 (16 %)			
1	200 (27 %)	27 (27 %)	9 (12 %)	164 (29 %)			
2	234 (31 %)	34 (33 %)	25 (35 %)	176 (31 %)			
≥3	214 (29 %)	36 (35 %)	38 (53 %)	140 (24 %)			
Underlying disease(s) [*n* (%)]							
Cardiovascular risk factors	415 (56 %)	66 (65 %)	48 (66 %)	302 (53 %)	0.87	3.1 × 10^−2^	4.3 × 10^−2^
Chronic heart failure	87 (12 %)	18 (18 %)	15 (21 %)	54 (9 %)	0.56	2.2 × 10^−2^	6.8 × 10^−3^
Chronic renal failure	118 (16 %)	20 (20 %)	18 (25 %)	80 (14 %)	0.46	0.17	2.1 × 10^−2^
Chronic respiratory disease	119 (16 %)	13 (13 %)	7 (10 %)	99 (17 %)	0.63	0.31	0.12
Neuropsychiatric disease	181 (24 %)	30 (30 %)	13 (18 %)	138 (24 %)	0.11	0.27	0.30
Cirrhosis	84 (11 %)	10 (10 %)	18 (25 %)	80 (14 %)	1.1 × 10^−2^	3.9 × 10^−2^	4.7 × 10^−3^
Solid tumor	109 (15 %)	12 (12 %)	14 (20 %)	83 (15 %)	0.20	0.54	0.29
Hematological malignancy	104 (14 %)	15 (15 %)	28 (38 %)	62 (11 %)	5.8 × 10^−4^	0.30	4.2 × 10^−8^
Immunodeficiency	188 (25 %)	32 (31 %)	47 (66 %)	109 (19 %)	6.7 × 10^−6^	7.9 × 10^−3^	1.4 × 10^−15^
McCabe score [*n* (%)]					1.6 × 10^−3^	0.55	6.7 × 10^−5^
No fatal underlying disease	413 (56 %)	63 (62 %)	27 (38 %)	323 (57 %)			
Underlying disease with expected life <5 years	239 (32 %)	28 (27 %)	23 (32 %)	188 (33 %)			
Underlying disease with expected life <1 year	91 (12 %)	11 (11 %)	21 (30 %)	59 (10 %)			
SAPS II (median [IQR])	40[29; 54]	42 [32; 51]	47 [37; 61]	40 [28; 54]	1.3 × 10^−2^	0.44	7.7 × 10^−4^
SOFA score (median [IQR])	5 [3; 9]	5 [3; 9]	7 [4; 10]	5 [2; 8]	1.8 × 10^−2^	0.19	1.4 × 10^−4^
Patients’ origin [*n* (%)]					5.2 × 10^−3^	0.66	1.5 × 10^−3^
Direct admission (ED; home)	400 (54 %)	59 (58 %)	26 (35 %)	316 (55 %)			
Secondary admission (ward, other hospitals)	343 (46 %)	43 (42 %)	45 (65 %)	254 (45 %)			
Main reason for admission [*n* (%)]					1.5 × 10^−4^	3.8 × 10^−8^	1.2 × 10^−2^
Acute respiratory failure	259 (35 %)	24 (24 %)	20 (28 %)	215 (38 %)			
Metabolic disorders	95 (13 %)	32 (31 %)	6 (8 %)	57 (10 %)			
Cardiac arrest	22 (3 %)	5 (5 %)	3 (4 %)	14 (2 %)			
Neurologic disorders	162 (22 %)	26 (25 %)	14 (19 %)	122 (21 %)			
Shock	172 (23 %)	15 (15 %)	28 (40 %)	129 (23 %)			
Other	33 (4 %)	0 (0 %)	0 (0 %)	33 (6 %)			

*ADE* adverse drug event, *ED* emergency department, *ICU* intensive care unit, *IQR* inter-quartile range, *SAPS II* Simplified Acute Physiology Score II, *SOFA* Sequential Organ Failure Assessment

### Unpreventable ADEs

ICU admissions attributed to unpreventable ADE affected patients with a significantly higher number of comorbidities, higher McCabe score and higher SAPS II than patients of the other groups (Table 1). In addition, admissions for shock were more frequent and patients originated more often from another department or another hospital. Examination of patients’ drug histories (Table 2) indicated that patients admitted for an unpreventable ADE-related admission had a significantly higher number of drugs recently introduced. Drugs involved in the unpreventable ADEs were more frequently prescribed at hospital, and nearly two-thirds were either antineoplastic and immunomodulating agents or drugs acting on blood and blood-forming organs (Table 3). Hospital mortality was significantly increased in patients with unpreventable ADE-related admission. Corresponding total LOS and hospital costs were significantly higher (Table 4).

**Table 2:** Drug history before intensive care unit admission

Characteristics	Total (*n* = 743)	Preventable ADE (*n* = 102)	Unpreventable ADE (*n* = 71)	Control (*n* = 570)	*P* value		
					Preventable–unpreventable	Preventable–control	Unpreventable–control
Number of drugs taken >1 month before ICU admission (median [IQR])	5 [2; 8]	5 [3; 8]	6 [4; 9]	5 [2; 8]	0.17	0.15	4.3 × 10^−3^
Number of drugs taken < 1 month before ICU admission (median [IQR])	3 [1; 5]	4 [1; 7]	8 [4; 12]	2 [0; 4]	6.0 × 10^−6^	1.6 × 10^−4^	2.2 × 10^−16^
Total number of drugs taken before ICU admission (median [IQR])	9 [5; 13]	11 [7; 15]	15 [12; 20]	8 [4; 11]	8.5 × 10^−7^	3.1 × 10^−5^	2.2 × 10^−16^
Number of drugs involved in the ADE^a^ [*n* (%)]					0.19	NA	NA
1	NA	33 (57 %)	29 (41 %)	NA			
2	NA	15 (26 %)	24 (34 %)	NA			
≥3	NA	10 (17 %)	18 (25 %)	NA			
Origin of prescriptions, *n* (%)					2.2 × 10^−16^	NA	NA
Hospital	NA	30 (29 %)	59 (83 %)	NA			
Community	NA	19 (19 %)	12 (17 %)	NA			
Other^b^	NA	53 (52 %)	0 (0 %)	NA			

*ADE* adverse drug event, *ICU* intensive care unit, *IQR* inter-quartile range, *NA* non-applicable

^a^Admissions due to non-compliance or drug underuse were excluded from the analysis (44 admissions in the preventable group) in the analysis of the item “number of drugs involved”

^b^Admissions due to self-medication, compliance problems or drug underuse

**Fig. 2:**
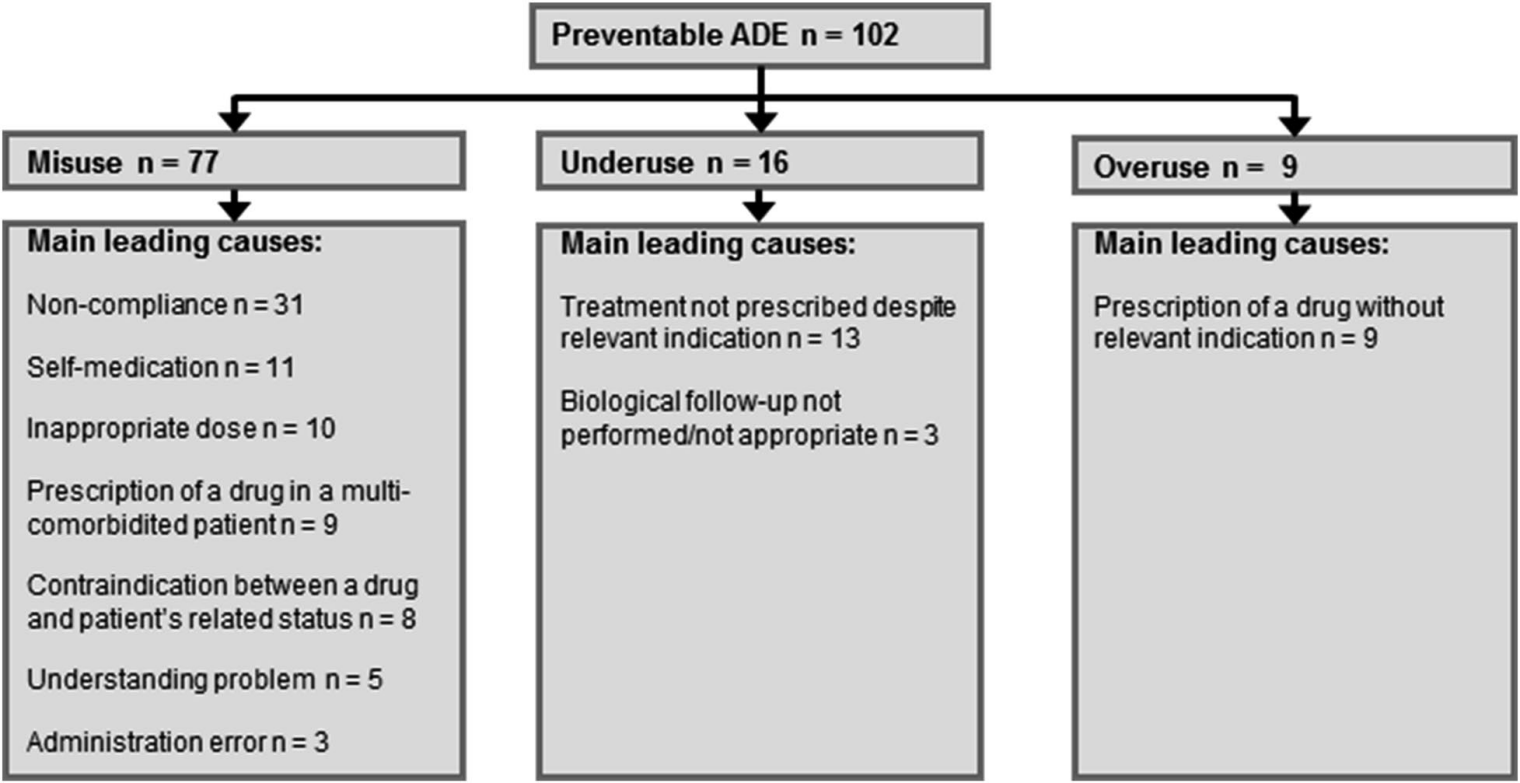
Analysis of preventable adverse drug events by main leading causes. *ADE* adverse drug event

**Table 3:** Drugs involved in preventable and unpreventable adverse drug events according to the anatomical therapeutic and chemical (ATC) classification (one ADE can be due to more than one drug)

Drugs classification according to ATC classification system	Preventable ADE [*n* (%)]	Unpreventable ADE [*n* (%)]	All ADE
Antineoplastic and immunomodulating agents	3 (3 %)	41 (41 %)	44 (20 %)
Cardiovascular system	36 (30 %)	8 (8 %)	44 (20 %)
Blood and blood-forming organs	20 (17 %)	23 (23 %)	43 (20 %)
Nervous system	30 (25 %)	13 (13 %)	43 (20 %)
Systemic hormonal preparation (except sex hormones and insulin)	9 (8 %)	7 (7 %)	16 (7 %)
Anti-infective for systemic use	7 (6 %)	5 (5 %)	12 (5 %)
Alimentary tract and metabolism	4 (3 %)	2 (2 %)	6 (3 %)
Musculoskeletal system	6 (5 %)	0 (0 %)	6 (3 %)
Others	4 (3 %)	1 (1 %)	5 (2 %)
Total	119 (100 %)	100 (100 %)	219 (100 %)

The distribution of ATC classes implied in preventable and unpreventable ADE was compared: *P* = 2.2 × 10^−13^

### Preventable ADEs

Preventable ADE-related and control groups shared a substantial number of patient baseline characteristics (Table 1). However, a significantly higher number of underlying diseases were observed in the preventable ADE-related group, and admissions for a metabolic disorder as well as renal replacement therapy were more frequent. Patients’ treatments prior to ICU admission included more drugs and more recently introduced drugs than those observed in the control group (Table 2). The most frequent types of drugs involved in preventable ADEs were cardiovascular drugs, nervous system drugs and drugs acting on blood and blood-forming organs (Table 3).

Unlike unpreventable ADE-related admissions, preventable ADE-related admissions did not significantly differ from control admissions in terms of hospital LOS and costs (Table 4). More importantly, the 102 preventable ADE-related admissions accounted for a total of 528 days of hospitalization in the ICU, equivalent to the requirement of 1.4 beds during the one-year study period. Moreover, the total costs related to these ICU periods of hospitalization observed during the year amounted to 747,651 €.

Considering the 102 ICU preventable ADE-related admissions, 77 were related to drug misuse, 16 to drug underuse and 9 to drug overuse (Fig. 2). The most frequent leading cause for drug misuse was non-compliance (30 % of ICU admissions related to preventable ADEs). The chronic diseases for which patients were not compliant with their medication were diabetes (*n* = 10), epilepsy (*n* = 6), cardiac chronic failure (*n* = 5), HIV (*n* = 4) and other conditions (*n* = 6). Reasons for non-compliance were psychiatric illness (*n* = 10), misunderstanding (*n* = 10), patient’s refusal (*n* = 8), cognitive impairment (*n* = 2) or omission (*n* = 1).

### Sensitivity analysis

Excluding from the analyses, the 26 admissions due to drug-related self-poisoning only resulted in the following changes: incidence estimate (among all admissions) of ADE-related, unpreventable ADE-related, and preventable ADE-related admissions increased from 23.3 to 24.1 % [95 % CI 20.4–26.5], from 9.6 to 9.9 % [95 % CI 7.9–12.3] and from 13.7 to 14.2 % [95 % CI 11.8–17.0], respectively; the hospital mortality in the unpreventable ADE and control groups was no longer significantly different; the frequency of the use of renal replacement therapy compared in the preventable ADE and control groups was no longer significantly different either (see Additional file 3: Table S3, Additional file 4: Table S4, Additional file 5: Table S5).

## Discussion

We have undertaken a one-year study analysis of admissions into a single ICU, focusing on ADEs. We have chosen a broad definition of ADE, including preventable and unpreventable ADE, as defined by Nebeker et al. [3] and the American National Roundtable on Health Care Quality [5].

We found an incidence of 23.3 % of ICU admissions due to ADE. Preventable ADEs accounted for 13.7 % and unpreventable ADEs for 9.6 % of ICU admissions. The incidence of ICU admissions due to ADE found in our study is the third highest incidence reported in the literature [7–18]. This high proportion of ICU admissions due to ADE can be explained by (1) the use of a broad definition of ADE, taking into account unpreventable ADE and all types of preventable ADE and (2) the profile of the hospital in which the study was conducted with oncology, hematology and hepatology departments which can be important providers of unpreventable ADE (in Table 3, note the high frequency of involvement of antineoplastic and immunomodulating agents in unpreventable ADE). Non-compliance was the leading cause of preventable ADEs. Importantly, the results indicate that preventable ADEs constitute a significant burden in ICU routine practice, with the requirement of 1.4 beds each day in the ICU, and a total corresponding direct cost of 747,651 euros for the duration of the study.

**Table 4:** Treatments and outcomes of intensive care unit admissions

Characteristics	Total (*n* = 743)	Preventable ADE (*n* = 102)	Unpreventable ADE (*n* = 71)	Control (*n* = 570)	*P* value		
					Preventable–unpreventable	Preventable–control	Unpreventable–control
Number of organ support(s) [*n* (%)]					0.34	0.37	0.28
0	312 (42 %)	48 (47 %)	25 (35 %)	239 (42 %)			
1	232 (31 %)	31 (30 %)	22 (31 %)	179 (31 %)			
2	128 (17 %)	12 (12 %)	13 (18 %)	103 (18 %)			
≥3	71 (10 %)	11 (11 %)	11 (16 %)	49 (9 %)			
Type of organ support [*n* (%)]							
Noninvasive ventilation	115 (15 %)	10 (10 %)	8 (11 %)	97 (17 %)	0.80	7.7 × 10^−2^	0.30
Invasive ventilation	270 (36 %)	30 (29 %)	29 (41 %)	211 (37 %)	0.14	0.15	0.52
Catecholamine	237 (32 %)	27 (26 %)	33 (46 %)	177 (31 %)	9.1 × 10^−3^	0.41	0.11
Renal replacement therapy	70 (9 %)	16 (16 %)	10 (14 %)	44 (8 %)	0.83	1.4 × 10^−2^	0.11
Massive blood transfusion (>1/2 blood volume)	26 (3 %)	7 (7 %)	6 (8 %)	13 (2 %)	0.77	2.1 × 10^−2^	1.3 × 10^−2^
Mortality during ICU admission [*n* (%)]^a^	125 (17 %)	14 (14 %)	18 (25 %)	93 (16 %)	7.2 × 10^−2^	0.56	6.7 × 10^−2^
Mortality during hospital admission[*n* (%)]^a^	161 (22 %)	17 (17 %)	25 (35 %)	119 (21 %)	6.7 × 10^−3^	0.42	9.8 × 10^−3^
Length of ICU stay (median [IQR])	4 [2; 7]	4 [2; 7]	4 [2; 9]	4 [2; 7]	0.15	0.49	0.26
Length of hospital stay (median [IQR])	13 [6; 29]	13 [5; 28]	23 [9; 48]	12 [5; 28]	5.4 × 10^−3^	0.47	1.2 × 10^−4^
Estimated costs (euros)of ICU admissions (median [IQR])	4651[2382; 10,298]	3688 [2562; 8705]	5802 [2425; 13,460]	4694 [2 342; 10 301]	7.4 × 10^−2^	0.52	0.11
Estimated costs (euros)of hospital admissions (median [IQR])	10,623 [5889; 18,698]	9015 [5823; 18,043]	13,933 [8429; 37,047]	10,204 [5749; 17,248]	8.5 × 10^−4^	0.97	5.2 × 10^−5^

*ADE* adverse drug event, *ICU* intensive care unit, *IQR* inter-quartile range

^a^Percentage of mortality based on the number of admissions

### Study limitations and strengths

Our study has some limitations. It is a monocentric study conducted in an ICU located in a hospital with departments specialized in oncology, hematology and hepatology. This might explain the observed high mortality rate and the length of hospital stay. We did not attempt to assess ADE-attributed mortality since it is almost impossible to assess respective contribution of underlying disease and drugs.

The study has also many strengths. To our knowledge, it is the first to examine in such great detail ICU admissions related to an ADE. The prospective screening of the present study allowed very accurate data collection of patient drug history and causality/preventability assessments. In order to minimize clinical judgment influence, inclusions in the ADE or in the control group were independently performed by a clinical pharmacist and an ICU physician, with a high inter-rater reliability. This reliability was much higher than that reported by others [12].

For causality assessment, 3 different scales were used [27–29]. As previously reported by Kane-Gill et al. [32] and Jolivot et al. [19], Karch and Lasagna and Naranjo scales are not tailored for ICU as ADE should be excluded if patient’s condition did not improve after drug dechallenge. Due to the severity of most of the cases, this condition cannot be fulfilled in ICU. Furthermore, rechallenge as suggested by Karch and Lasagna is unethical, since ADE was the leading cause of admission. Nevertheless, applying these scales allowed some comparison with international studies. The third scale is better suited for ICU [27] but has only been used in French studies. Hence, in order to be comprehensive, we applied Kane-Gill et al. recommendations [33] and used these 3 scales of assessment.

The sensitivity analysis indicates that considering admissions attributed to drug-related self-poisonings in the control group or excluding such admissions from the analyses had a very moderate impact on the study results. This can be explained by the small proportion of these admissions in the control group (*n* = 26) and their associated relatively low severity.

### Unpreventable ADEs

Unpreventable ADEs accounted for 41 % of ICU admissions related to ADE. In this group, patients took more drugs before ICU admission than in the control group.

The patient case mix which involved immunocompromised and onco-hematological patients might explain both this high proportion (with frequent events due to antineoplastic and immunomodulating agents and frequent admissions for shock, especially septic shock) [34] and the higher severity score observed in the unpreventable ADE group. The proportion of unpreventable ADEs such as sepsis during febrile aplasia was particularly high, as previously reported by Nazer et al. [17].

### Preventable ADEs

Preventable ADEs accounted for 59 % of ICU admissions related to ADE. Previous studies reported corresponding estimates within a range of 17.5–85.7 % [7–18, 35], and adding our study to these would rank the present study estimate at 4/9. Cost, LOS and mortality were similar to those of control group, suggesting that these preventable ADEs are as life-threatening as other events leading to ICU admission. This also suggests that implementation of corrective action might be cost-saving.

ICU admissions due to preventable ADEs involved recently introduced treatments (prescribed for less than a month) and mainly drugs for the cardiovascular (30 %) and nervous systems (25 %). Nervous system drugs were also one of the most frequent classes of drugs prescribed in France in 2013 [36]. This raises the important question of the relative potential of drugs to induce ADE. Further studies dealing with this issue should be performed.

Eight studies have focused on the determination of the leading causes of ICU admissions due to preventable ADE [7–10, 12, 14, 17, 18]. Similar leading causes to that found in our study were reported (in variable proportions): drug misuse (dose error, inadequate follow-up, drug interaction, administration error and contraindication), underuse (absence of prophylaxis) and overuse (inappropriate drug). However, the present study is the first to date to provide a comprehensive description of non-compliance and self-medication as leading causes of ICU admissions. The fact that patients in the preventable ADE group came more often from home or the Emergency Department than those in the unpreventable ADE group is in line with the study of Heaton et al. which reported that non-compliance substantially required Emergency Department visits [37]. In our study, medication non-compliance was the principal leading cause of ICU admissions due to preventable ADE (30 %), showing that adherence failure might also generate severe outcomes and a significant burden for the healthcare system.

A third of the medication non-compliance-related admissions (*n* = 10) resulted from misunderstanding problems from patients with chronic disease who did not understand that they had to take their chronic treatments for life. Low patient adherence is a substantial problem in therapy. As shown by our study, it can lead to ICU admission. Causes of non-adherence are various and include poor patient education, level of understanding, lack of explanations given by the doctor or lack of pharmacist intervention. Many interventions were tested to enhance therapy adherence (e.g., inducing behavior change in patients, face-to-face information delivery by pharmacists, connexion between daily routine and drug administration). However, the authors of a recent meta-analysis conclude that a number of clinical trials describing interventions intended to enhance patient adherence lack proving their efficacy [38].

Reducing the substantial burden of ICU admissions due to preventable ADE is an issue of concern. Future research should explore how various interventions might favorably impact this burden, such as physician training, drug conciliation by a pharmacist or therapeutic education.

## Conclusions

In this observational study, we report that 23.3 % of ICU admissions were caused by an ADE, with 59 % of them related to a preventable ADE. Nearly a third of the corresponding preventable ADEs were due to non-compliance. Our study provides a strong rationale for undertaking future studies to explore the impact of potential corrective actions aiming at reducing ADE-related ICU admissions.

## Additional files

10.1186/s13613-016-0109-9 Organ disorders associated with drugs implicated in adverse drug events.
10.1186/s13613-016-0109-9 Definitions and classifications used in the study.
10.1186/s13613-016-0109-9 Baseline characteristics and outcomes of intensive care unit admissions if excluding self-poisoning-related admissions.
10.1186/s13613-016-0109-9 Drug history before ICU admission if excluding self-poisoning-related admissions.
10.1186/s13613-016-0109-9 Treatments and outcomes of intensive care unit admissions if excluding self-poisoning-related admissions.

## Abbreviations

ADE: adverse drug event
ADR: adverse drug reaction
ATC: anatomical therapeutic and chemical
ICD: International Classification of Diseases
ICU: intensive care unit
LOS: length of stay
ME: medication error
MeSH: medical subheadings
SAPS: Simplified Acute Physiology Score
SmPC: Summary Product Characteristics
SOC: System Organ Class
SOFA: Sequential Organ Failure Assessment
WHO: World Health Organization
