# Supplementary material for: An observational study of adult admissions to a medical ICU due to adverse drug events
Source: Ann Intensive Care. 2016 Feb 2;6:9. doi: 10.1186/s13613-016-0109-9 (PMC4735088; doi:10.1186/s13613-016-0109-9)
Supplement: Supplementary file 1 — 10.1186/s13613-016-0109-9 Organ disorders associated with drugs implicated in adverse drug events. [file 13613_2016_109_MOESM1_ESM.docx]

Additional Table S1: Organ disorders associated with drugs implicated in adverse drug events (according to ATC classification system). One adverse drug event could be due to more than one drug.

ATC = Anatomical Therapeutic and Chemical, NSAID = Nonsteroidal Anti-Inflammatory Drug.

| **Drugs classification according to ATC classification system** | **ADE classification according to System Organ Class (SOC) terms, n (%)** | | | | | | | | | | | | | | |
| --- | --- | --- | --- | --- | --- | --- | --- | --- | --- | --- | --- | --- | --- | --- | --- |
|  | Blood and lymphatic system disorders | Cardiac disorders | Endocrine disorders | Gastrointestinal disorders | Hepatobiliary disorders | Immune system disorders | Infections and infestations | Injury, poisoning and procedural complications | Metabolism and nutrition disorders | Nervous system disorders | Psychiatric disorders | Renal and urinary disorders | Respiratory, thoracic and mediastinal disorders | Vascular disorders | **Total**  **(n, %)** |
| Antineoplastic and immunomodulating agents | 18 (40%) | 2 (5%) | 0 (0)% | 0 (0)% | 1 (2%) | 1 (2%) | 17 (39%) | 0 (0)% | 2 (5%) | 0 (0)% | 0 (0)% | 2 (5%) | 1 (2%) | 0 (0)% | **44 (20%)** |
| Blood and blood forming organs (anticoagulants and antiaggregants) | 0 (0%) | 5 (12%) | 0 (0%) | 4 (9%) | 0 (0%) | 0 (0%) | 1 (2%) | 7 (16%) | 0 (0%) | 7 (16%) | 0 (0%) | 0 (0%) | 1 (2%) | 18 (43%) | **43 (20%)** |
| Nervous system  (sedatives/hypnotics and analgesics) | 0 (0)% | 10 (23%) | 0 (0)% | 1 (2%) | 3 (7%) | 0 (0)% | 0 (0)% | 0 (0)% | 1 (2%) | 20 (48%) | 4 (9%) | 0 (0)% | 4 (9%) | 0 (0)% | **43 (20%)** |
| Systemic hormonal preparations (corticosteroids) | 0 (0)% | 0 (0)% | 4 (26%) | 0 (0)% | 1 (6%) | 0 (0)% | 8 (50%) | 0 (0)% | 2 (12%) | 0 (0)% | 0 (0)% | 0 (0)% | 0 (0)% | 1 (6%) | **16 (7%)** |
| Anti-infective for systemic use | 1 (8%) | 1 (8%) | 0 (0)% | 0 (0)% | 0 (0)% | 2 (17%) | 0 (0)% | 0 (0)% | 0 (0)% | 3 (25%) | 1 (8%) | 4 (34%) | 0 (0)% | 0 (0)% | **12 (5%)** |
| Alimentary tract and metabolism (including oral antidiabetics) | 0 (0%) | 0 (0%) | 1 (17%) | 0 (0%) | 0 (0%) | 0 (0%) | 0 (0%) | 0 (0%) | 1 (17%) | 2 (32%) | 0 (0%) | 1 (17%) | 0 (0%) | 1 (17%) | **6 (3%)** |
| Musculo-skeletal system (including NSAID) | 0 (0)% | 0 (0)% | 0 (0)% | 0 (0)% | 0 (0)% | 0 (0)% | 4 (67%) | 0 (0)% | 0 (0)% | 0 (0)% | 0 (0)% | 2 (33%) | 0 (0)% | 0 (0)% | **6 (3%)** |
| Other | 0 (0)% | 1 (20%) | 0 (0)% | 0 (0)% | 0 (0)% | 0 (0)% | 0 (0)% | 0 (0)% | 0 (0)% | 0 (0)% | 0 (0)% | 3 (60%) | 1 (20%) | 0 (0)% | **5 (2%)** |
| **Total, n (%)** | **19 (9%)** | **26 (12%)** | **5 (2%)** | **5 (2%)** | **5 (2%)** | **3 (1%)** | **30 (14%)** | **7 (3%)** | **10 (5%)** | **32 (15%)** | **10 (5%)** | **37 (17%)** | **7 (3%)** | **23 (10%)** | **219 (100%)** |
