# Supplementary material for: An observational study of adult admissions to a medical ICU due to adverse drug events
Source: Ann Intensive Care. 2016 Feb 2;6:9. doi: 10.1186/s13613-016-0109-9 (PMC4735088; doi:10.1186/s13613-016-0109-9)
Supplement: Supplementary file 2 — 10.1186/s13613-016-0109-9 Definitions and classifications used in the study. [file 13613_2016_109_MOESM2_ESM.docx]

Additional Table S2: Definitions and classifications used in the study.

1. **Adverse drug event**: any injury from medical intervention related to a drug.[[1](#_ENREF_1)]
2. **Adverse drug reaction**: any noxious and unintended effect of a drug occurring at doses normally used in man for the prophylaxis, diagnosis, or therapy of the disease, or for the modification of physical function.[[2](#_ENREF_2)]
3. **Medication error**: any preventable event that may cause or lead to inappropriate medication or patient harm while the medication is in the control of the health care professional, patient or consumer.[[1](#_ENREF_1)]
4. **Underuse**: failure to detect diseases or to use proven effective treatments.[[3](#_ENREF_3)]
5. **Overuse**: situations in which potential for harm exceeded the possible benefit.[[3](#_ENREF_3)]
6. **Misuse**: corresponds to an appropriate treatment with occurrence of a preventable complication.[[3](#_ENREF_3)]
7. **Schumock and Thornton modified preventability scale [**[**4**](#_ENREF_4)**]**

Answer “Yes” or “No” to each of the following questions.

Answering “Yes” to one or more of the following questions suggests that the ADR in question may indeed have been preventable,

- Was the drug involved in the ADR *not* considered appropriate for the patient’s clinical condition?
- Was the dose, route, and the frequency of administration *not* appropriate for the patient’s age, weight and disease state?
- Was required therapeutic drug monitoring or other necessary laboratory test *not* performed?
- Was there a history of allergy or previous reaction to the drug?
- Was a drug interaction **(contraindicated)** involved in the reaction?
- Was a toxic serum drug level documented?
- Was poor compliance involved in the reaction?
- **Was an appropriate prophylactic treatment *not* prescribed?**

**Bold terms were added for our study from the initial scale**

1. **French method of causality assessment [**[**5**](#_ENREF_5)**]**

**Table de décision combinant les critères chronologiques (C).**

*Chaque médicament pris par le malade doit être imputé successivement et de manière indépendante*

| **Administration du médicament** | **Délai d’apparition de l’évènement** | | |
| --- | --- | --- | --- |
|  | très suggestif | compatible | incompatible |
| **Arrêt du médicament** | Réadministration du médicament | |  |
|  | R+ R(0) R(-) | R+ R(0) R(-) |  |
| **Evolution suggestive :** régression de l’évènement coïncidant bien avec cet arrêt | C_3_ C_3_ C_1_ | C_3_ C_2_ C_1_ | C_0_ |
| **Evolution non concluante :** régression paraissant au contraire plutôt spontanée ou provoquée par un traitement symptomatique non sépcifique réputé efficace sur ces troubles, ou évolution inconnue, ou recul insuffisant ou lésions de type irréversible | C_3_ C_2_ C_1_ | C_3_ C_2_ C_1_ | C_0_ |
| **Evolution non suggestive :** absence de régression d’un évènement de type réversible (ou régression complète malgré la poursuite du médicament) | C_1_ C_1_ C_1_ | C_1_ C_1_ C_1_ | C_1_ |

R+ : positive : l’évènement récidive, R0 : non faite ou non évaluable, R- : négative : l’évènement ne récidive pas, C_3_ : chronologie vraisemblable, C_2_ : plausible, C_1_ douteuse, C_0_ : paraissant exclure le rôle du médicament

**Table de décision combinant les critères sémiologiques (S)**

| **Sémiologie (clinique ou paraclinique)** | **Evocatrice du rôle de ce médicament (et/ou facteur très favorisant bien validé)** | **Autres éventualités sémiologiques** |
| --- | --- | --- |
| **Autre explication non médicamenteuse** | examen complémentaire spécifique fiable | |
|  | **L+ L0 L-** | **L+ L0 L-** |
| absente (après bilan approprié) | **S_3_ S_3_  S_1_** | **S_3_ S_2_  S_1_** |
| possible (non recherchée ou présente) | **S_3_ S_2_  S_1_** | **S_3_ S_1_  S_1_** |

L+ : test de laboratoire positif, L0 : test non disponible pour le couple évènement-médicament considéré, L- : test négatif, S_3_ : sémiologie vraisemblable, S_2_: plausible, S_1_ douteuse

**Table de décision de l’imputabilité intrinsèque. Cette imputabilité est établie par croisement des scores chronologiques (C) et sémiologiques (S)**

| **Chronologie** | **Sémiologie** | | |
| --- | --- | --- | --- |
|  | **S_1_** | **S_2_** | **S_3_** |
| **C_0_** | **I_0_** | **I_0_** | **I_0_** |
| **C_1_** | **I_1_** | **I_1_** | **I_2_** |
| **C_2_** | **I_1_** | **I_2_** | **I_3_** |
| **C_3_** | **I_3_** | **I_3_** | **I_4_** |

**I_4_:** Imputabilité intrinsèque **très vraisemblable I_3_: vraisemblable**

**I_2 :_ plausible I_1_: douteuse I0 : paraissant exclue**

1. **Naranjo method for causality assessment [**[**6**](#_ENREF_6)**]**

| To assess the adverse drug reaction, please answer the following questionnaire and give the pertinent score | | | | |
| --- | --- | --- | --- | --- |
|  | Yes | No | Do not know | Score |
| 1. Are there previous conclusive reports on this reaction? | +1 | 0 | 0 |  |
| 2. Did the adverse event appear after the suspected drug was administered? | +2 | -1 | 0 |  |
| 3. Did the adverse reaction improve when the drug was discontinued or a specific antagonist was administered? | +1 | 0 | 0 |  |
| 4. Did the adverse reaction reappear when the drug was readministered? | +2 | -1 | 0 |  |
| 5. Are there alternative causes (other than the drug) that could on their own have caused the reaction? | -1 | +2 | 0 |  |
| 6. Did the reaction reappear when a placebo was given? | -1 | +1 | 0 |  |
| 7. Was the drug detected in the blood (or other fluids) in concentrations known to be toxic? | +1 | 0 | 0 |  |
| 8. Was the reaction more severe when the dose was increased, or less severe when the dose was decreased? | +1 | 0 | 0 |  |
| 9. Did the patient have a similar reaction to the same or similar drugs in any previous exposure | +1 | 0 | 0 |  |
| 10. Was the adverse event confirmed by any objective evidence | +1 | 0 | 0 |  |
|  |  |  | Total score |  |

Interpretation :

Score :

- >9: definite
- 5-8: probable
- 1-4: possible
- Doubtfull ≤ 0

1. **Karch and Lasagna method for causality assessment [**[**7**](#_ENREF_7)**]**

| Appropriate interval between agent-event | No | Yes | Yes | Yes | Yes | Yes | Yes | Yes | Yes | Yes |
| --- | --- | --- | --- | --- | --- | --- | --- | --- | --- | --- |
| Known reaction to agent | *___* | No | No | Yes | Yes | Yes | Yes | Yes | Yes | Yes |
| Event reasonably explained by clinical state or other (nondrug) therapies | *___* | Yes | No | Yes | Yes | No | No | No | No | No |
| Dechallenge attempted | *___* | *___* | *___* | *___* | *___* | No | Yes | Yes | Yes | Yes |
| Improve with dechallenge | *___* | *___* | *___* | *___* | *___* | *___* | No | Yes | Yes | Yes |
| Rechallenge attempted | *___* | *___* | *___* | *___* | *___* | *___* | *___* | No | Yes | Yes |
| Relapse on rechallenge | *___* | *___* | *___* | Yes | No | *___* | *___* | *___* | No | Yes |
| Definite |  |  |  |  |  |  |  |  |  | X |
| Probable |  |  |  | X |  | X |  | X |  |  |
| Possible |  |  |  |  | X |  |  |  | X |  |
| Conditional |  |  | X |  |  |  |  |  |  |  |
| Unrelated | X | X |  |  |  |  | X |  |  |  |

1. Nebeker JR, Barach P, Samore MH. Clarifying adverse drug events: a clinician's guide to terminology, documentation, and reporting. Annals of internal medicine. 2004;140(10):795-801.

2. World Health Organisation. The importance of pharmacovigilance - Safety Monitoring of medical products. Geneva. 2002. p. 40.

3. Chassin MR, Galvin RW. The urgent need to improve health care quality. Institute of Medicine National Roundtable on Health Care Quality. Jama. 1998;280(11):1000-5.

4. Schumock GT, Thornton JP. Focusing on the preventability of adverse drug reactions. Hospital pharmacy. 1992;27(6):538.

5. Begaud B, Evreux JC, Jouglard J, Lagier G. [Imputation of the unexpected or toxic effects of drugs. Actualization of the method used in France]. Therapie. 1985;40(2):111-8.

6. Naranjo CA, Busto U, Sellers EM, Sandor P, Ruiz I, Roberts EA et al. A method for estimating the probability of adverse drug reactions. Clinical pharmacology and therapeutics. 1981;30(2):239-45.

7. Karch FE, Lasagna L. Toward the operational identification of adverse drug reactions. Clin Pharmacol Ther. 1977;21(3):247-54.
