# Supplementary material for: An observational study of adult admissions to a medical ICU due to adverse drug events
Source: Ann Intensive Care. 2016 Feb 2;6:9. doi: 10.1186/s13613-016-0109-9 (PMC4735088; doi:10.1186/s13613-016-0109-9)
Supplement: Supplementary file 3 — 10.1186/s13613-016-0109-9 Baseline characteristics and outcomes of intensive care unit admissions if excluding self-poisoning-related admissions. [file 13613_2016_109_MOESM3_ESM.docx]

Additional Table S3: Baseline characteristics and outcomes of intensive care unit admissions if excluding self-poisoning-related admissions

ED: Emergency Department; ICU: Intensive Care Unit; IQR: Interquartile Range; SAPS II: Simplified Acute Physiology Score II; SOFA: Sequential Organ Failure Assessment.

| **Characteristics** | **Total**  **n = 717** | **Preventable ADE**  **n = 102** | **Unpreventable ADE**  **n = 71** | **Control**  **n = 544** | **P value** | | |
| --- | --- | --- | --- | --- | --- | --- | --- |
|  |  |  |  |  | Preventable-Unpreventable | Preventable- Control | Unpreventable-Control |
| **Age** (median [IQR]) | 65 [51;78] | 63 [47;77] | 65 [55;75] | 66 [51;78] | 0.60 | 0.51 | 0.95 |
| **Gender** (males/females; sex ratio) | 417/300; 1.39 | 50/52; 0.96 | 43/28; 1.53 | 324/220; 1.47 | 0.16 | 5.0 x 10^-2^ | 0.90 |
| **Number of underlying disease(s)**, n (%) |  |  |  |  | 1.2 x 10^-2^ | 5.1 x 10^-3^ | 1.26 x 10^-8^ |
| 0 | 95 (13%) | 5 (5%) | 0 (0%) | 90 (17%) |  |  |  |
| 1 | 183 (26%) | 27 (27%) | 9 (12%) | 147 (27%) |  |  |  |
| 2 | 228 (32%) | 34 (33%) | 25 (35%) | 170 (31%) |  |  |  |
| ≥ 3 | 211 (29%) | 36 (35%) | 38 (53%) | 137 (25%) |  |  |  |
| **Underlying disease(s)**, n (%) |  |  |  |  |  |  |  |
| Cardiovascular risk factors | 407 (57%) | 66 (65%) | 48(66%) | 294 (54%) | 0.87 | 5.1 x 10^-2^ | 5.7 x 10^-2^ |
| Chronic heart failure | 87 (12%) | 18 (18%) | 15 (21%) | 54 (10%) | 0.56 | 3.8 x 10^-2^ | 8.6 x 10^-3^ |
| Chronic renal failure | 114 (16%) | 20 (20%) | 18 (25%) | 76 (14%) | 0.46 | 0.17 | 2.1 x 10^-2^ |
| Chronic respiratory disease | 116 (16%) | 13(13%) | 7 (10%) | 96 (18%) | 0.63 | 0.25 | 0.13 |
| Neuropsychiatric disease | 157 (22%) | 30 (30%) | 13 (18%) | 114 (21%) | 0.11 | 7.0 x 10^-2^ | 0.75 |
| Cirrhosis | 84 (12%) | 10 (10%) | 18 (25%) | 68 (12%) | 1.1 x 10^-2^ | 6.0 x 10^-2^ | 5.8 x 10^-3^ |
| Solid tumour | 108 (15%) | 12 (12%) | 14 (20%) | 82 (15%) | 0.20 | 0.45 | 0.30 |
| Haematological malignancy | 104 (15%) | 15 (15%) | 28 (38%) | 62 (11%) | 5.8 x 10^-4^ | 0.33 | 1.0 x 10^-7^ |
| Immunodeficiency | 188 (26%) | 32 (31%) | 47(66%) | 109 (20%) | 6.7 x 10^-6^ | 1.3 x 10^-2^ | 8.5 x 10^-15^ |
| **Mac Cabe Score,** n (%) |  |  |  |  | 1.6 x 10^-3^ | 0.34 | 1.7 x 10^-4^ |
| No fatal underlying disease | 387 (54%) | 63 (62%) | 27 (38%) | 297 (55%) |  |  |  |
| Underlying disease with expected life < 5 years | 239 (33%) | 28 (27%) | 23 (32%) | 188 (35%) |  |  |  |
| Underlying disease with expected life <1 year | 91 (13%) | 11 (11%) | 21 (30%) | 59 (10%) |  |  |  |
| **SAPSII** (median [IQR]) | 40[29; 54] | 42 [32;51] | 47 [37;61] | 40 [28; 54] | 1.3 x 10^-2^ | 0.43 | 7.8 x 10^-4^ |
| **SOFA score** (median [IQR]) | 5 [2; 9] | 5 [3; 9] | 7 [4;10 ] | 5 [2; 8] | 1.8 x 10^-2^ | 0.18 | 1.5 x 10^-4^ |

Additional Table S2 (continued)

| **Characteristics** | **Total**  **n = 717** | **Preventable ADE**  **n = 102** | **Unpreventable ADE**  **n = 71** | **Control**  **n = 544** | **P value** | | |
| --- | --- | --- | --- | --- | --- | --- | --- |
|  |  |  |  |  | Preventable-Unpreventable | Preventable- Control | Unpreventable-Control |
| **Patients’ origin**, n (%) |  |  |  |  | 5.2 x 10^-3^ | 0.45 | 5.3 x 10^-3^ |
| Direct admission (ED; Home) | 374 (52%) | 59 (58%) | 26 (35%) | 290 (53%) |  |  |  |
| Secondary admission (ward, other hospitals) | 343 (48%) | 43 (42%) | 45 (65%) | 254 (47%) |  |  |  |
| **Main reason for admission,** n (%) |  |  |  |  | 1.5 x 10^-4^ | 1.1 x 10^-8^ | 1.1 x 10^-2^ |
| Acute respiratory failure | 259 (36%) | 24 (24%) | 20 (28%) | 215 (40%) |  |  |  |
| Metabolic disorders | 94 (13%) | 32 (31%) | 6 (8%) | 56 (10%) |  |  |  |
| Cardiac arrest | 22 (3%) | 5 (5%) | 3 (4%) | 14 (2%) |  |  |  |
| Neurologic disorders | 138 (19%) | 26 (25%) | 14 (19%) | 98 (18%) |  |  |  |
| Shock | 171 (24%) | 15 (15%) | 28 (40%) | 129 (24%) |  |  |  |
| Other | 33 (5%) | 0 (0%) | 0 (0%) | 33 (6%) |  |  |  |
