# Supplementary material for: An observational study of adult admissions to a medical ICU due to adverse drug events
Source: Ann Intensive Care. 2016 Feb 2;6:9. doi: 10.1186/s13613-016-0109-9 (PMC4735088; doi:10.1186/s13613-016-0109-9)
Supplement: Supplementary file 4 — 10.1186/s13613-016-0109-9 Drug history before ICU admission if excluding self-poisoning-related admissions. [file 13613_2016_109_MOESM4_ESM.docx]

Additional Table S4: Drug history before intensive care unit admission if excluding self-poisoning-related admissions

* Admissions due to non compliance or drug underuse were excluded from the analysis (44 admissions in the preventable group) in the analysis of the item "number of drugs involved ** Admissions due to self-medication, to compliance problems or drug underuse

ADE = adverse drug events; ICU = intensive care unit; IQR = Interquartile Range; NA = nonapplicable

| **Characteristics** | **Total**  **n = 717** | **Preventable ADE**  **n = 102** | **Unpreventable ADE**  **n = 71** | **Control**  **n = 544** | **P value** | | |
| --- | --- | --- | --- | --- | --- | --- | --- |
|  |  |  |  |  | Preventable-Unpreventable | Preventable Control- | Unpreventable- Control |
| **Number of drugs taken > 1 month before ICU admission** (median [IQR]) | 5 [2;8] | 5 [3;8] | 6 [4;9] | 5 [2;8] | 0.17 | 0.15 | 3.8 x 10^-3^ |
| **Number of drugs taken < 1 month before ICU admission** (median [IQR]) | 3 [1 ;5] | 4 [1;7] | 8 [4 ;12] | 2 [1 ;4] | 6.0 x 10^-6^ | 3.9 x 10^-4^ | 2.2 x 10^-16^ |
| **Total number of drugs taken before ICU admission** (median [IQR]) | 9 [5;13] | 11 [7;15] | 15 [12;20] | 8 [4;11] | 8.5 x 10^-7^ | 4.9 x 10^-5^ | 2.2 x 10^-16^ |
| **Number of drugs involved in the ADE^*^,** n (%) |  |  |  |  | 0.19 | NA | NA |
| 1 | NA | 33 (57%) | 29 (41%) | NA |  |  |  |
| 2 | NA | 15 (26%) | 24 (34%) | NA |  |  |  |
| ≥ 3 | NA | 10 (17%) | 18 (25%) | NA |  |  |  |
| **Origin of prescriptions,** n (%) |  |  |  |  | 2.2 x 10^-16^ | NA | NA |
| Hospital | NA | 30 (29%) | 59 (83%) | NA |  |  |  |
| Community | NA | 19 (19%) | 12 (17%) | NA |  |  |  |
| Other**^**^** | NA | 53 (52 %) | 0 (0%) | NA |  |  |  |
