# Supplementary material for: An observational study of adult admissions to a medical ICU due to adverse drug events
Source: Ann Intensive Care. 2016 Feb 2;6:9. doi: 10.1186/s13613-016-0109-9 (PMC4735088; doi:10.1186/s13613-016-0109-9)
Supplement: Supplementary file 5 — 10.1186/s13613-016-0109-9 Treatments and outcomes of intensive care unit admissions if excluding self-poisoning-related admissions. [file 13613_2016_109_MOESM5_ESM.docx]

Additional Table S5: Treatments and outcomes of intensive care unit admissions if excluding self-poisoning-related admissions

ADE: Adverse Drug Event; ICU: Intensive Care Unit; IQR: Interquartile Range.

* Percentage of mortality based on the number of admissions

| **Characteristics** | **Total**  **n = 717** | **Preventable ADE**  **n = 102** | **Unpreventable ADE**  **n = 71** | **Control**  **n = 544** | **P value** | | |
| --- | --- | --- | --- | --- | --- | --- | --- |
|  |  |  |  |  | Preventable-Unpreventable | Preventable- Control | Unpreventable-Control |
| **Number of organ support(s)**, n (%) |  |  |  |  | 0.34 | 0.43 | 0.33 |
| 0 | 303 (42%) | 48 (47%) | 25 (35%) | 230 (42%) |  |  |  |
| 1 | 220 (31%) | 31 (30%) | 22 (31%) | 167 (31%) |  |  |  |
| 2 | 123 (17%) | 12 (12%) | 13 (18%) | 98 (18%) |  |  |  |
| ≥ 3 | 71 (10%) | 11 (11%) | 11 (16%) | 49 (9%) |  |  |  |
| **Type of organ support**, n (%) |  |  |  |  |  |  |  |
| Non-invasive ventilation | 114 (16%) | 10 (10%) | 8 (11%) | 96 (18%) | 0.80 | 5.8 x 10^-2^ | 0.24 |
| Invasive ventilation | 253 (35%) | 30 (29%) | 29 (41%) | 194 (36%) | 0.14 | 0.26 | 0.43 |
| Catecholamine | 234 (33%) | 27 (26%) | 33 (46%) | 174 (32%) | 9.1 x 10^-3^ | 0.30 | 2.2 x 10^-2^ |
| Renal replacement therapy | 69 (10%) | 16 (16%) | 10 (14%) | 43 (8%) | 0.83 | 2.3 x 10^-2^ | 0.11 |
| Massive blood transfusion (>1/2 blood volume) | 26 (4%) | 7 (7%) | 6 (8%) | 13 (2%) | 0.77 | 2.6 x 10^-2^ | 1.5 x 10^-2^ |
| **Mortality during ICU admission**, n (%)* | 125 (17%) | 14 (14%) | 18 (25%) | 93 (17%) | 7.2 x 10^-2^ | 0.47 | 0.10 |
| **Mortality during hospital admission**, n (%)* | 161 (22%) | 17 (17%) | 25 (35%) | 119 (22%) | 6.7 x 10^-3^ | 0.29 | 1.7 x 10^-2^ |
| **Length of ICU stay** (median [IQR]) | 4 [2;7] | 4 [2;7] | 4 [2;9] | 4 [2;7] | 0.15 | 0.38 | 0.33 |
| **Length of hospital stay** (median [IQR]) | 13 [6;30] | 13 [5;28] | 23 [9;48] | 13 [6;28] | 5.4 x 10^-3^ | 0.73 | 3.6 x 10^-4^ |
| **Estimated costs (euros) of ICU admissions** (median [IQR]) | 4,771 [2428;10462] | 3,688 [2,562; 8,705] | 5,802 [2425;13460] | 4,824 [2422;10503] | 7.4 x 10^-2^ | 0.37 | 0.16 |
| **Estimated costs (euros) of hospital** **admissions** (median [IQR]) | 10,861 [6,243;19,049] | 9015 [5,823;18043] | 13,933 [8,429;37,047] | 10,687 [6,173;17,947] | 8.5 x 10^-4^ | 0.60 | 2.0 x 10^-4^ |
